# Identification of an uncharacterized gene as a mitochondrial methionine tRNA synthetase in *Caenorhabditis elegans*

**DOI:** 10.1093/g3journal/jkaf298

**Published:** 2025-12-08

**Authors:** Bharat Vivan Thapa, Mohit Das, David I Taylor, James P Held, Maulik R Patel

**Affiliations:** Department of Biological Sciences, Vanderbilt University, Nashville, TN 37232, United States; Department of Metabolism and Nutritional Programming, Van Andel Institute, Grand Rapids, MI 49503, United States; Department of Biological Sciences, Vanderbilt University, Nashville, TN 37232, United States; Department of Biological Sciences, Vanderbilt University, Nashville, TN 37232, United States; Department of Metabolism and Nutritional Programming, Van Andel Institute, Grand Rapids, MI 49503, United States; Department of Metabolism and Nutritional Programming, Van Andel Institute, Grand Rapids, MI 49503, United States

**Keywords:** mitochondria, mtDNA, tRNAs, metionine tRNA synthetase, mitochondrial unfolded protein response, *Caenorhabditis elegans*, translation, mars-2, WormBase

## Abstract

Aminoacyl-tRNA synthetases (aaRSs) are essential for translation, as they charge tRNA molecules with their corresponding amino acids. Alterations in aaRSs can significantly disrupt both cytosolic and mitochondrial translation. Through a forward genetic screen for mitochondrial unfolded protein response (UPR^mt^) activators in *Caenorhabditis elegans*, we identified a missense mutation (P447V) in the previously uncharacterized gene Y105E8A.20, which encodes for a methionine tRNA synthetase (MetRS). Here, we characterize the UPR^mt^ induction by Y105E8A.20, which we call *mars-2*, and demonstrate that the P447V allele is a loss-of-function mutation. Furthermore, we show that impaired *mars-2* activity leads to reduced mitochondrial-encoded protein abundance, depletion of mitochondrial membrane potential, fragmented mitochondrial morphology, and mild developmental delay, although the animals remain viable. Hence, this hypomorphic mars-2(P447V) strain provides a valuable tool for studying mitochondrial translation and understanding how aaRSs are involved in mitochondrial homeostasis.

## Introduction

Mitochondria are multifaceted organelles involved in a wide range of essential processes, including energy production, intracellular signaling, and programmed cell death (Chen et al. 2023; Suomalainen and Nunnari 2024). This semiautonomous organelle has its own genome. In *Caenorhabditis elegans*, the mitochondrial genome encodes 36 genes, including essential protein subunits of the mitochondrial respiratory complexes, mitochondrial rRNAs, and mitochondrial tRNAs (Okimoto et al. 1992). The mitochondrial genome is transcribed as a single polycistronic RNA, which is subsequently processed and then mRNAs are translated within mitochondria by specialized mito-ribosomes (Ojala et al. 1981). While mitochondrial tRNAs are encoded within mitochondria, they must be charged with their corresponding amino acids to locally translate mitochondrial DNA-encoded proteins. This charging process is carried out by aminoacyl-tRNA synthetases (aaRSs), which are encoded by the nuclear genome and imported into the mitochondria (Sissler et al. 2017). aaRSs are highly specialized enzymes involved in covalently attaching amino acids to their cognate tRNAs. Given their essentiality across eukaryotes, bacteria, and archaea (Wolf et al. 1999; Giegé and Springer 2016), mutations in aaRS genes have detrimental consequences including being linked to Charcot–Marie–Tooth (CMT) disease (Antonellis et al. 2003; Jordanova et al. 2006; Latour et al. 2010; Boczonadi et al. 2018; Wei et al. 2019), tumor formation (Arif et al. 2009; Han et al. 2012; Kim et al. 2014), and death (Shin et al. 2008). Moreover, recent studies show that mutations in aaRS can alter tRNA levels, which in turn regulate the production of tRNA-derived fragments and contribute to diverse disease phenotypes (Torres et al. 2019).

Although mitochondrial aaRS mutations are associated with several diseases (Edvardson et al. 2007; Scheper et al. 2007; Tang et al. 2015), the mechanisms by which these mutations affect mitochondrial function and lead to pathology are insufficiently explored. Dysfunctional aaRS activity can directly affect mitochondrial protein synthesis causing mitochondrial stress (Konovalova and Tyynismaa 2013; Maffezzini et al. 2019). Mitochondrial quality control pathways, including the mitochondrial unfolded protein response (UPR^mt^), are activated in response to mitochondrial stress (Zhao et al. 2002). UPR^mt^ upregulates nuclear-encoded chaperones and proteases to preserve mitochondrial functionality (Melber and Haynes 2018; Shpilka and Haynes 2018). In *C. elegans*, this response is mediated by the transcription factor ATFS-1, which in healthy cells is imported to the mitochondria (Nargund et al. 2012). However, when mitochondria are stressed, a decrease in mitochondrial protein import disrupts ATFS-1 mitochondrial import (Haynes et al. 2013), resulting in ATFS-1 nuclear accumulation and subsequent transcription of UPR^mt^-associated genes. Transcriptional reporters, such as the *hsp-6p::GFP* strain (one of the UPR^mt^-associated genes), have been developed to monitor UPR^mt^ activation in live animals (Yoneda et al. 2004).

Here we report that a missense mutation in a previously uncharacterized methionine tRNA synthetase gene in *C. elegans* activates UPR^mt^. Using CRISPR-Cas9, we confirmed the causality of this point mutation and demonstrated that it represents a loss-of-function allele. Systematic analysis of the 2 *mars-2* isoforms suggests that the mitochondrial-specific loss of MARS-2 leads to UPR^mt^. Furthermore, we show that hypomorphic *mars-2(P447V)* mutant animals exhibit reduced mitochondrial-encoded protein abundance, decreased mitochondrial membrane potential, mitochondrial fragmentation, and mild developmental delay relative to wild-type. Our characterization of MARS-2 as a mitochondrial tRNA synthetase that is required for proper mitochondrial function provides novel insight into mitochondrial quality control and the strains we generated promise to be important tools to further study mitochondrial translation and homeostasis.

## Materials and methods

### Worm strains

All the strains used in this study are listed in Table 1.

**Table 1.** List of strains used in this study.

Genotype	Source	Strain name
*C. elegans* wild-type	CGC	N2
zcIs13[hsp-6p::GFP] V	CGC	GL347
mptsi1, zcIs13V; mpt139	Held et al. (2024)	MRP649
mars-2(mpt213[P447V]) I	This study	MRP1019
mars-2(mpt215[P447V]) I; zcIs13V	This study	MRP1021
mars-2(mpt227[M1A]) I +/−; zcIs13V	This study	MRP1042
mars-2(syb10542[M119A]) I	SUNY Biotech	PHX10542
mars-2(syb10543[M119A]) I	SUNY Biotech	PHX10543
mars-2(syb10542[M119A]) I; zcIs13V	This study	MRP1144
mars-2(syb10543[M119A]) I; zcIs13V	This study	MRP1145
mars-2(syb10569[mars-2::GFP]) I	SUNY Biotech	PHX10569
mars-2(syb10782[$\frac{\mathrm{mars}-2(M1A)::\mathrm{GFP}}{\mathrm{hIn}1(\mathrm{unc}-54[h1040])}$]) I	SUNY Biotech	PHX10782
mars-2(syb10666[mars-2(M119A)::GFP]) I	SUNY Biotech	PHX10666
mars-2(syb10806[$\frac{\mathrm{mars}-2(P447V)::\mathrm{GFP}}{\mathrm{hIn}1(\mathrm{unc}-54[h1040])}$]) I	SUNY Biotech	PHX10806
scpl-4(wbm108[TIMM-50::scarlet]) V	Valera-Alberni et al. (2024)	WBM1688
mars-2(syb10569[mars-2::GFP]) I; scpl-4(wbm108[TIMM-50::scarlet]) V	This study	MRP1146
mars-2(syb10666[mars-2(M119A)::GFP]) I; scpl-4(wbm108[TIMM-50::scarlet]) V	This study	MRP1147
nuo-6(qm200) I	CGC	MQ1333
mars-2(mpt213[P447V]) I; scpl-4(wbm108[TIMM-50::scarlet]) V	This study	MRP1143
zcIs4[hsp-4p::GFP] V	CGC	SJ4005 mars-2(mpt213[P447V]) I
zcIs4 V	This study	MRP1209

### Worm maintenance

All worms were raised at 20 °C on nematode growth media (NGM) seeded with OP50  *Escherichia coli* bacteria.

### CRISPR/Cas9 gene editing

CRISPR was performed as previously described (Dokshin et al. 2018) using Alt-R S.p. Cas9 Nuclease V3 (IDT #1081058), tracrRNA (IDT #1072532), and using *dpy-10* as a co-injection marker (Paix et al. 2015). The crRNA sequence used for introducing P447V mutation in *mars-2* was TCAATCAAGTCAACCATGGA and ssODN sequence: TGAAAGAAGCGAATCGATTATTTCAATCAAGTCAATCATGGAAAGAAATTGATGAAAAACGGCTGAAAAGTCTACTTTT. The crRNA sequence used for introducing M1A mutation in *mars-2* was 5′-CGCTTTTCATGAATCCTTGG-3′ and ssODN sequence: 5′-cgattgattaattcacttttttttttgcgcttttcGCTAATCCATGGAGATTTTTCGTGAGAAAATCGAGTACATTTGTCACTTC-3′. The Cas9::tracrRNA::crRNA complexes and repair templates were microinjected into the N2 strain to obtain *mpt213* and *mpt227*, respectively. The *dpy-10* co-injection marker was then outcrossed using a wild-type (N2, RRID: WBStrain00000001) strain.

### Genetic crosses

The genetic crosses were performed by crossing 5 larval stage 4 (L4) hermaphrodites of a strain to 15 heterozygous males of another strain (these were first obtained by crossing L4 hermaphrodites of the strain with wild-type males). An appropriate number of F1 generation L4 hermaphrodites were cloned out of the cross plate and allowed to have self-progeny. An appropriate number of F2 progeny were cloned and genotyped for allele of interest once they had progeny. The genotyping was performed using IDT primers and NEB enzymes below:

For genotyping P447V mutation in *mars-2*, Forward-GGAAATGGTCGAGGAGAGCAGAG, Reverse-CCTCCAGAATAGCTTCCAAATCGGG, and NcoI cuts wild-type amplicon only. For genotyping M1A mutation in *mars-2*, Forward-CAGGAATTGAAGGAGCTAAATTCTGCAAAG, Reverse-CGAAATTAGGGATTATCAGACGCAAGTTCC, and NcoI-HF cuts mutant amplicon only.

### Fluorescence microscopy

Zeiss Axio Zoom V16 stereo zoom microscope was used for imaging fluorescent UPR^mt^ reporter and TMRE stain in whole animals. The worms were mounted on 2% agarose pads on microscopic slides and immobilized using 1 µL of 100 mM levamisole (ThermoFisher #AC187870100). A coverslip was used to avoid the worms from drying. Nikon Spinning Disk confocal microscope at 63× magnification was used for imaging mitochondrial morphology using SCPL-4::wrmScarlet strain. Andor Spinning Disk Confocal Dragonfly 620 microscope was used for imaging mitochondria and MARS-2::GFP for co-localization experiment.

### Image analysis

The brightfield and fluorescent animal images were analyzed using the FIJI application. The outline of each worm was traced as the region of interest (ROI) on the brightfield snapshot. The “Measure” feature under “Analyze” tab was used for measuring the area of the animal and its fluorescence intensity (calculated by summing all the pixel values within the ROI and dividing by the total number of pixels in that area) in the fluorescence snapshot. For confocal microscopy images, the colocalization analysis and Manders coefficients were obtained by using built-in “Coloc 2” Plugin under “Analyze > Colocalization” tab. For mitochondria morphology analysis, Mitochondria Analyzer plugin (Chaudhry et al. 2020) was used to generate mitochondrial masks for maximum intensity projected images and the same plugin was used to calculate mean aspect ratio and mean branch length of the mitochondria.

### TMRE protocol

TMRE plates were made by adding 500 µL of 1 mM TMRE (ThermoFisher #T669) in M9 buffer (stock TMRE solution—0.5 M in DMSO) on pre-seeded stand NGM plates with OP50 and allowed to dry overnight in the dark. L4 animals were transferred to TMRE plates and incubated for 16 h, followed by a transfer to standard seeded NGM plates for an hour to remove any nonspecific TMRE signal from the cuticle and intestinal lumen. These animals were then imaged using Zeiss Axio fluorescent microscope.

### Egg hatching assay

Hundred embryos per genotype were transferred onto individual OP50 seeded NGM plates. These embryos were incubated for 16 h at 20 °C, and the number of hatched larval animals for wild-type and mutant strains were counted after 16 h. The hatching assay was performed in duplicates.

### Development assay

Hundred embryos per genotype were transferred onto individual OP50-seeded NGM plates. These embryos were incubated for 36 h at 20 °C. After 36-h time point, wild-type and mutant animals were counted, and assessed for the larval or adult stages every 12 h (till 96-h time point) by picking them onto new seeded NGM plates. Larval animals smaller than L4 were identified by their smaller size, animals at L4 stage were identified by the characteristic “semilunar” structure of the developing vulva under brightfield, and adult animals were identified by the presence of embryos within the transparent animal or disappearance of the developing vulva structure.

### Western blotting

Hundred animals per genotype were picked into 18 µL of M9 buffer. 12 µL of 10% SDS was added to each sample, animals were lysed at 95 °C for 10 min, and each sample was pipetted up and down 5 times to mix. 4 × Laemmli buffer (BioRad #161-0737) was mixed with 2-mercaptoethanol in 3:1 ratio and 10 µL of the mixture was added to each sample. Samples were denatured at 95 °C for 5 min and pipetted up and down 5 times to mix, followed by a quick spin to get rid of the debris. 18 µL of the denatured protein lysate was loaded onto Mini-PROTEAN TGX Gel (BioRad #4561095). The gel was run for 30 min at 100 V, then 50 min for 130 V in 1 × TGS running buffer (BioRad #1610732). Next, the gel was equilibrated in Trans-Blot Turbo transfer buffer (BioRad #10026938) and transferred to activated equilibrated LF PVDF Membrane (BioRad #10026934) for 7 min at 2.5 A/25 V on Trans-Blot Turbo transfer system. Post transfer, the membrane was blocked for 2 h at room temperature in 5% milk in TBST solution. Post blocking, membrane was incubated at 4 °C overnight with primary antibodies. Anti-MTCO1 mouse monoclonal (1:1,000)—abcam #ab14705 and Anti-NDUFS3 mouse monoclonal (1:750)—abcam #ab14711. Post primary incubation, membrane was washed 3 times for 5 min each with 1× TBST solution. Membranes were then incubated with polyclonal Peroxidase AffiniPure Donkey Anti-mouse IgG (Jackson ImmunoResearch # 715-035-150) at a 1:5,000 dilution. The membranes were again washed 3 times with 1× TBST for 5 min each. After the final wash step, membranes were incubated for 5 min in Clarity Western ECL substrate (BioRad #1705060) and immediately imaged on BioRad Chemidoc MP imager. Both colorimetric and chemiluminescent channels were used for imaging the blot at low and high exposure times, and band intensities were quantified using FIJI.

### RNA interference

RNAi was performed by shifting embryos to NGM plates seeded with RNAi bacteria, as described previously (Held et al. 2022). *E. coli*  HT115 carrying specific RNAi clones was grown overnight from a single colony in 2 mL of LB supplemented with 50 μg/mL ampicillin. To make 16 RNAi plates, 50 mL of LB supplemented with 50 μg/mL ampicillin and inoculated with 500 μL of the overnight culture, then incubated while shaking at 37 °C for 4 to 5 h (to an OD_550–600_ of ∼0.8). Next, to induce expression of the double-stranded RNA, cultures were supplemented with an additional 50 mL of LB supplemented with 50 μg/mL ampicillin and 4 mM IPTG and then continued to incubate while shaking at 37 °C for 4 h. Following incubation, bacteria were pelleted by centrifugation at 3,900 rpm for 6 min. Supernatant was decanted and pellets were gently resuspended in 4 mL of LB supplemented with 8 mM IPTG. 250 μL of resuspension was seeded onto standard NGM plates containing 1 mM IPTG. Plates were left to dry overnight and then used within 2 weeks. Bacterial RNAi strains were from Ahringer RNAi Feeding Library, grown from single colony and identity confirmed by Sanger sequencing. *Y105E8A.20* (Y105E8A.20), *atfs-1* (ZC376.7), *dve-1* (ZK1193.5).

### Statistical analysis

For whole animal fluorescence analysis, a sample size of 24 was used in the study and each animal was considered a biological replicate. The *P*-values under 0.05 were considered significant while *P*-values above 0.05 were considered nonsignificant (ns—nonsignificant, *P < 0.05, **P < 0.01, ***P < 0.001, ****P < 0.0001). All statistical analysis was performed using Prism 10, and the information on specific statistical test can be found in the figure legend.

## Results

### Proline residue is a highly conserved amino acid residue in MARS-2

**Fig. 1.**
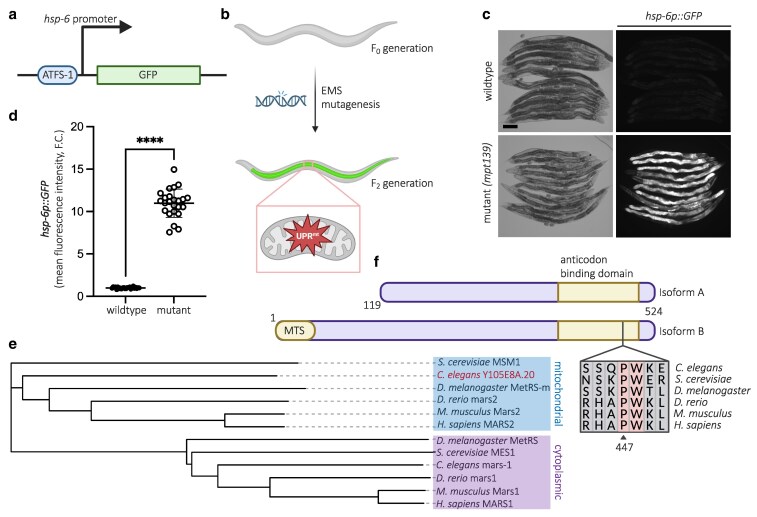
Phylogenetic analysis show that P447 is a highly conserved amino acid residue across species. a) Gene transcription diagram depicting the expression of *hsp-6p::GFP* transcriptional reporter. b) Schematic for the forward genetic screen to identify activators of mitochondrial unfolded protein response. c) Representative brightfield and corresponding fluorescent micrographs showing UPR^mt^ reporter (*hsp-6p::GFP*) activation in day 2 (D2) adult wild-type and mutant (*mpt139)* animals. d) Scatter plot showing fluorescence intensity quantification of the UPR^mt^ reporter in wild-type and mutant animals normalized to the reporter intensity in wild-type animals (*n* = 24; mean and SD shown; unpaired *t*-test with Welch's correction). e) Phylogenetic tree illustrating evolutionary relationships between species based on their mitochondrial and cytoplasmic methionine tRNA synthetase protein sequences (Y105E8A.20 highlighted in red). f) Pictorial depiction of the 2 isoforms of MARS-2 protein, along with the conserved proline residue (P447) in the anticodon binding domain of the protein in *C. elegans*. Scale bar—200 µm. GFP, green fluorescent protein; MTS, mitochondrial targeting sequence; FC, fold change. Created in BioRender. Thapa, B. (2025). https://BioRender.com/782jpb9

In a previous study (Held et al. 2024), we used the *hsp-6p::GFP* strain (Fig. 1a) to perform a forward genetic screen to identify unknown UPR^mt^-activating mutations in *C. elegans* (Fig. 1b). This screen yielded a strain (mpt139) with variants including a missense mutation (P447V) in the gene *Y105E8A.20*, which encodes a methionine tRNA synthetase (Fig. 1c and 1d). Phylogenetic analysis of protein sequences from cytoplasmic and mitochondrial methionine tRNA synthetases (MARS1 and MARS2, respectively) across various species revealed that Y105E8A.20 protein in *C. elegans*—hereafter referred to as MARS-2—shares greater similarity with MARS2 proteins compared to MARS1 proteins in other species (Fig. 1e). Furthermore, protein sequence alignment using CLUSTAL (Madeira et al. 2024) shows that the 447th amino acid residue is highly conserved across species (Fig. 1f).

### P447V mutation in MARS-2 activates mitochondria-specific unfolded stress response

**Fig. 2.**
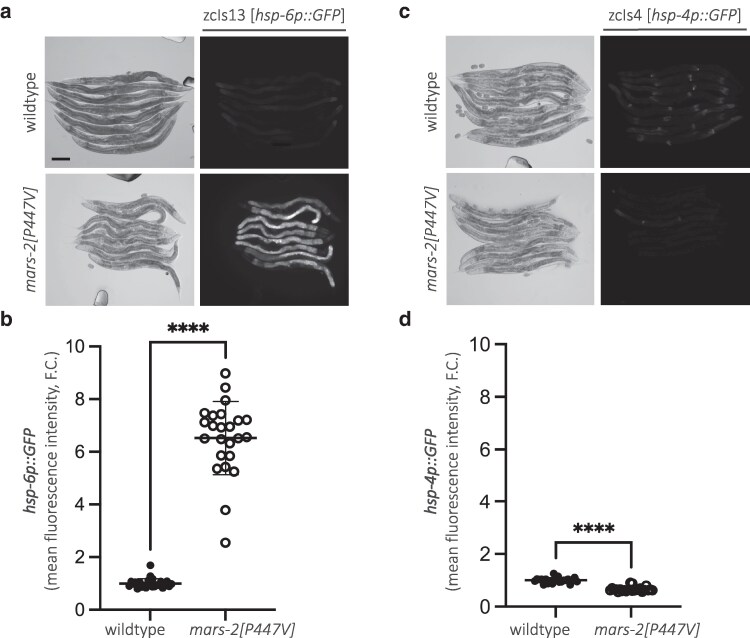
P447V is the causal mutation in MARS-2 that induces mitochondrial unfolded protein response. a) Representative brightfield and corresponding fluorescent micrographs showing UPR^mt^ reporter (*hsp-6p::GFP*) activation in D2 adult wild-type and *mars-2[P447V]* animals. b) Scatter plot showing fluorescence intensity quantification of the UPR^mt^ reporter in wild-type and *mars-2[P447V]* animals normalized to the reporter intensity in wild-type animals. c) Representative brightfield and corresponding fluorescent micrographs showing UPR^ER^ reporter (*hsp-4p::GFP*) induction in D2 adult wild-type and *mars-2[P447V]* animals. d) Scatter plot showing fluorescence intensity quantification of the UPR^ER^ reporter in wild-type and *mars-2[P447V]* animals normalized to the reporter intensity in wild-type animals (*n* = 24; mean and SD shown; unpaired *t*-test with Welch's correction). Scale bar—200 µm.

To confirm that the *mars-2(P447V)* mutation is causal in activating UPR^mt^, we introduced this missense mutation into the *zcIs13V* background (*hsp-6p::GFP* strain) using CRISPR-Cas9 gene editing. Two independent clones carrying the desired mutation were recovered and validated via Sanger sequencing. Fluorescent imaging of whole animals shows that the P447V mutant exhibited a 7-fold increase in UPR^mt^ reporter activation compared to wild-type animals (Fig. 2a and 2b), confirming the mutation's causative role in inducing UPR^mt^. Additionally, we assessed whether mars-2(P447V) causes unfolded protein stress outside of the mitochondria. We utilized a transcriptional reporter for endoplasmic reticulum (ER) stress, *zcIs4V* (*hsp-4p::GFP*), and observed no induction of UPR^ER^ in the mars-2(P447V) strain compared to wild-type animals (Fig. 2c and 2d).

### P447V mutation in MARS-2 induces mitochondrial unfolded protein response via ATFS-1 signaling

**Fig. 3.**
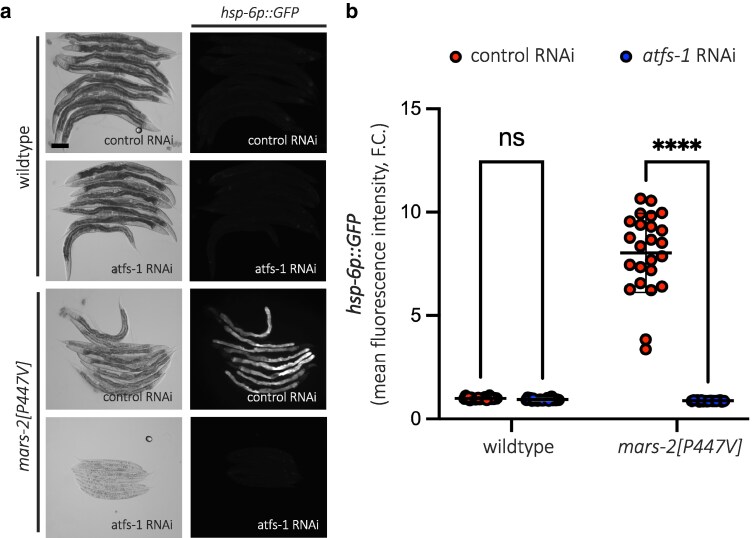
MARS-2(P447V)-mediated mitochondrial unfolded protein response is ATFS-1 dependent. a) Representative brightfield and corresponding fluorescent micrographs showing UPR^mt^ reporter activation in D2 adult wild-type and *mars-2[P447V]* animals upon control and *atfs-1* RNAi. b) Scatter plot showing fluorescence intensity quantification of the UPR^mt^ reporter in wild-type and *mars-2[P447V]* animals upon control and *atfs-1* RNAi normalized to the reporter intensity in wild-type animals on control RNAi (*n* = 18 in *mars-2[P447V]* on *atfs-1* RNAi, *n* = 24 others; mean and SD shown; 2-way ANOVA with Sidak's multiple comparisons test). Scale bar—200 µm.

Previous studies have shown that ATFS-1 plays a central role in inducing UPR^mt^ in *C. elegans* (Nargund et al. 2012; Haynes et al. 2013). To determine whether the P447V mutation in MARS-2 activates UPR^mt^ through this pathway, we performed RNAi-mediated knockdown of *atfs-1* in the mutant strain. This completely abolished reporter activation (Fig. 3a and 3b), confirming that UPR^mt^ activation in the mutant strain occurs via ATFS-1. Interestingly, animals carrying the P447V mutation arrested upon *atfs-1* knockdown (Fig. 3a). This likely reflects the inability to mount an adequate stress response in the absence of ATFS-1, consistent with other reports that ATFS-1 is protective against mitochondrial dysfunction (Nargund et al. 2012).

Additional studies have also implicated DVE-1 as an accessory regulator of UPR^mt^ in *C. elegans* (Haynes et al. 2007; Tian et al. 2016). Knockdown of *dve-1* by RNAi partially attenuates UPR^mt^ activation by *mars-2(P447V)* mutation (Supplementary Fig. 1a and 1b). Collectively, these findings demonstrate that the P447V mutation in *mars-2* gene drives UPR^mt^ activation via the ATFS-1-dependent pathway.

### P447V mutation in MARS-2 is a loss-of-function mutation

**Fig. 4.**
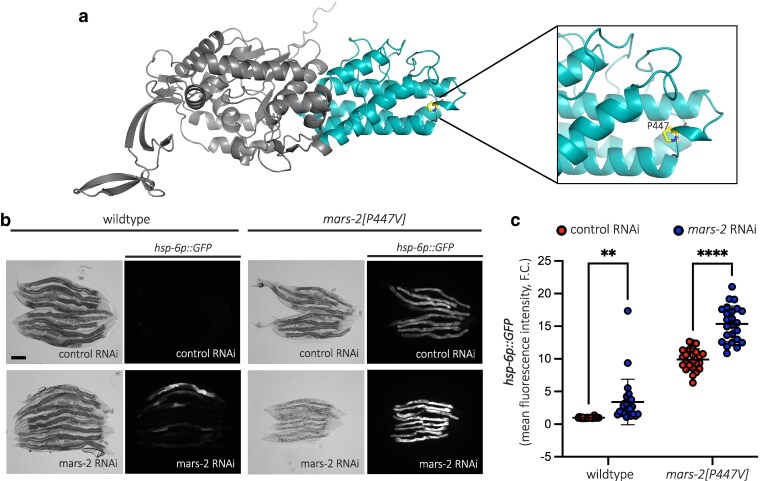
P447V mutation in MARS-2 is a loss-of-function mutation. a) Predicted structure of *C. elegans* MARS-2 protein (isoform B) using AlphaFold (gray). The anticodon-binding domain shown in cyan (pIDDT > 90, very high confidence), and 447th proline residue is marked in the inset. b) Representative brightfield and corresponding fluorescent micrographs showing UPR^mt^ reporter activation in D2 adult wild-type and *mars-2[P447V]* animals upon control and *mars-2* RNAi. c) Scatter plot showing fluorescence intensity quantification of the UPR^mt^ reporter in wild-type and *mars-2[P447V]* animals upon control and *mars-2* RNAi normalized to the reporter intensity in wild-type animals on control RNAi (*n* = 24; mean and SD shown; 2-way ANOVA with Sidak's multiple comparisons test). Scale bar—200 µm.

Next, we asked what effect the P447V mutation has on the protein. *C. elegans* MARS-2 protein structure was predicted using AphaFold (Jumper et al. 2021) and InterPro domain analysis (Blum et al. 2025) identified the P447V mutation within the anticodon-binding domain of the MARS-2 protein (Fig. 4a), a region critical for recognizing the anticodon loop of methionine tRNA. Given that mutations in the anticodon-binding domain of other aaRSs are often associated with reduced enzymatic activity (James et al. 2006; Scheper et al. 2007), we hypothesized that P447V mutation in *mars-2* represents a loss-of-function mutation. To test this, we performed RNAi targeting *mars-2* transcripts in wild-type and the mutant strain. Second-generation RNAi of *mars-2* in wild-type animals induced UPR^mt^ (Fig. 4b and 4c). Furthermore, *mars-2* RNAi in P447V mutant animals exacerbated UPR^mt^ activation and growth defects (Fig. 4b and 4c), consistent with P447V being a loss-of-function mutation.

### Loss of mitochondrial isoform of MARS-2 activates robust mitochondrial unfolded protein response

**Fig. 5.**
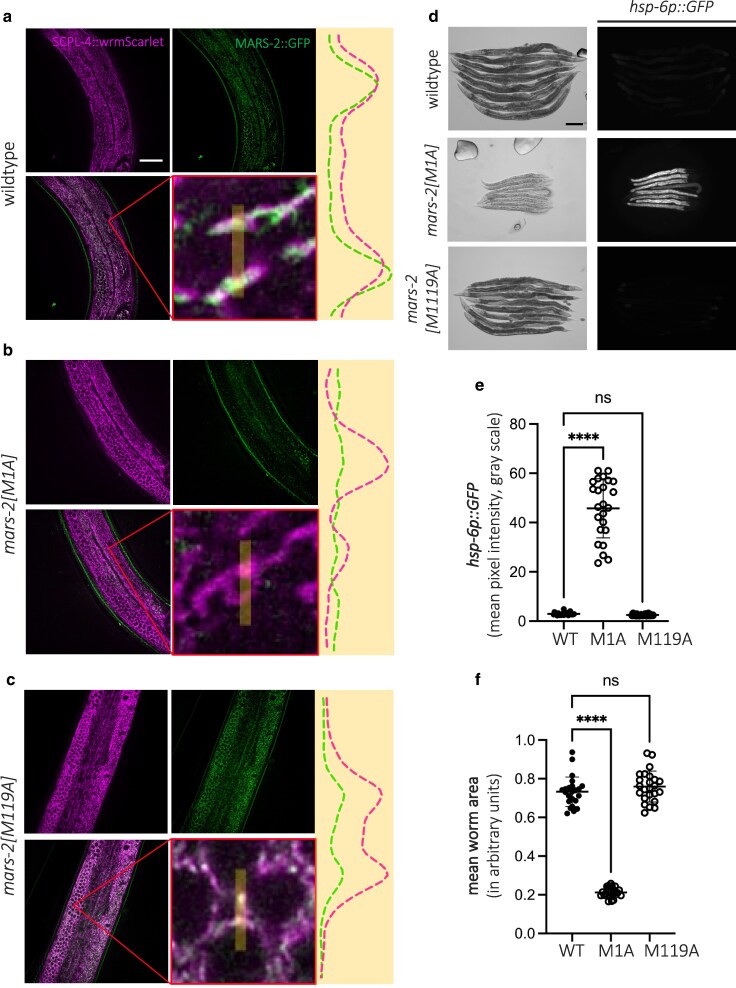
Loss of the mitochondrial isoform of MARS-2 by mutating the first methionine residue to alanine activates the mitochondrial unfolded protein response. Representative confocal images showing mitochondrial fluorescence using the WBM1688 (SCPL-4::wrmScarlet) strain (top-left), MARS-2::GFP signal (top-right), merged image of both channels (bottom-left), and a magnified inset highlighting the overlap between mitochondrial and MARS-2::GFP signal (bottom-right). A line intensity profile illustrating colocalization across a 5-µm line in the inset is displayed adjacent to the MARS-2::GFP and inset panels. Images are shown for D2 adult wild-type (a), *mars-2[M1A]* (b), and *mars-2[M119A]* (c) animals. Scale bar—20 µm. d) Representative brightfield and corresponding fluorescent micrographs showing UPR^mt^ reporter activation in D2 adult wild-type, *mars-2[M1A],* and *mars-2[M119A]* animals. Scale bar—200 µm. e) Scatter plot showing fluorescence intensity quantification of the UPR^mt^ reporter in wild-type, *mars-2[M1A]*, and *mars-2[M119A]* animals. f) Scatter plot depicting outline area quantification of wild-type, *mars-2[M1A]*, and *mars-2[M119A]* animals (*n* = 24; mean and SD shown; 1-way ANOVA with Dunnett's multiple comparisons test).

The *mars-*2 gene is predicted to encode 2 isoforms: isoform A (406 aa) and isoform B (524 aa). Analysis using MitoFates software (Fukasawa et al. 2015) predicts a mitochondrial targeting sequence (MTS) within the N-terminal region of isoform B, which is absent in isoform A. To assess the subcellular localization of these isoforms, we tagged endogenous MARS-2 at its C-terminus with GFP and crossed it into the WBM1688 (SCPL-4::wrmScarlet) strain, which labels mitochondria (Valera-Alberni et al. 2024). Confocal imaging revealed strong colocalization of MARS-2::GFP with mitochondrial signals, with a Mander's coefficient of 0.85 (Fig. 5a and Supplementary Table 1).

To further dissect the isoform-specific localization, we used CRISPR-Cas9 gene editing in the endogenously GFP-tagged strain to individually mutate the first and 119th methionine residues to alanine, thereby generating isoform B-specific (*mars-2(M1A)*) and isoform A-specific (*mars-2(M119A)*) knockout strains, respectively. In the *mars-2(M1A)* strain, animals exhibited developmental arrest, and heterozygotes were therefore used for imaging. These animals have markedly reduced GFP signal with minimal overlap between mitochondrial and MARS-2::GFP fluorescence (apart from autofluorescence from the gut granules in the green channel) (Fig. 5b), yielding a Mander's coefficient of 0.39 (Supplementary Table 1). This finding indicates that isoform B localizes to mitochondria and that the M1A mutation disrupts its production. In contrast, disruption of isoform A (*mars-2(M119A)*) did not affect mitochondrial colocalization of MARS-2::GFP (Fig. 5c and Supplementary Table 1), suggesting that isoform A is nonmitochondrial.

Having established the localization patterns of the 2 isoforms, we next generated and crossed the isoform-specific knockout strains with the *zcIs13* reporter strain to assess UPR^mt^ activation. Animals homozygous for the M1A mutation exhibit robust UPR^mt^ induction, whereas those harboring the M119A mutation (2 independent clones) did not (Fig. 5d and 5e and Supplementary Fig. 2), indicating that the mitochondrial isoform (isoform B) of *mars-2* is essential for maintaining mitochondrial homeostasis.

Together, these findings suggest that the P447V mutation represents a hypomorphic loss-of-function allele of *mars-2* that partially compromises its mitochondrial function but remains viable, in contrast to the severe growth defects and developmental arrest observed in the M1A mutants (Fig. 5d and 5f).

### P447V mutation in *mars-2* impairs mitochondrial translation, leading to mitochondrial membrane potential loss, hatching defects, and developmental delay

**Fig. 6.**
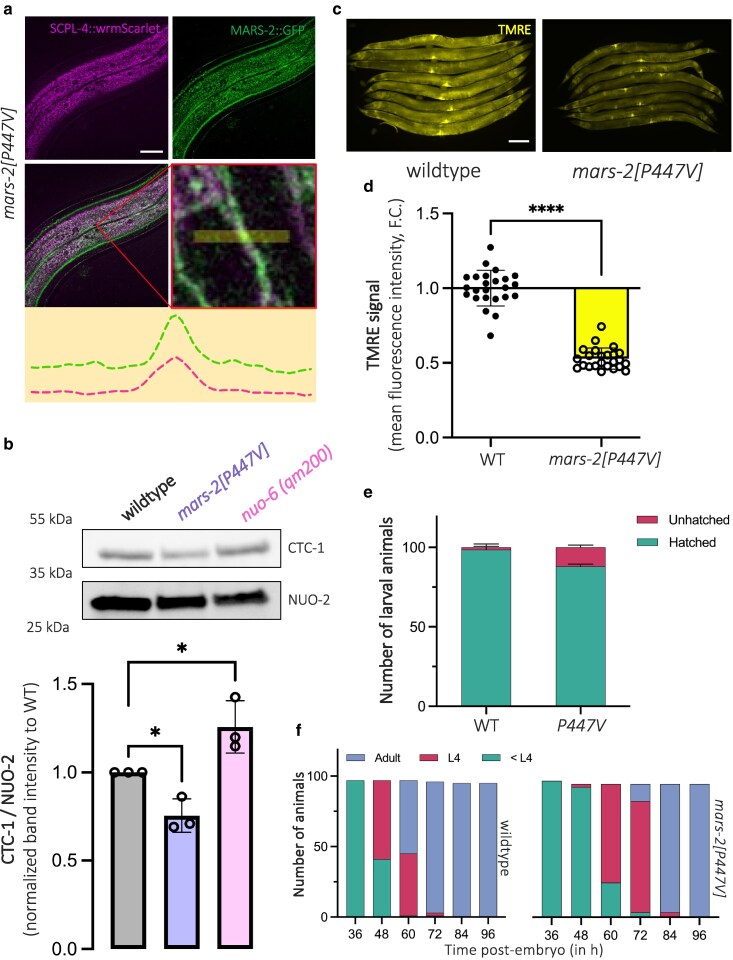
P447V mutation in *mars-2* impairs mitochondrial translation, leading to loss of mitochondrial membrane potential, hatching defects, and developmental delay. a) Representative confocal images of D2 adult *mars-2[P447V]* animals showing mitochondrial fluorescence using the WBM1688 (SCPL-4::wrmScarlet) strain (top-left), MARS-2::GFP signal (top-right), merged image of both channels (bottom-left), and a magnified inset highlighting the overlap between mitochondrial and MARS-2::GFP signal (bottom-right). A line intensity profile illustrating colocalization across a 5 µm line in the inset is displayed adjacent to the merged and inset panels. Scale bar—20 µm. b) Western blot showing chemiluminescent detection of mitochondrially encoded CTC-1 and the nuclear-encoded NUO-2 proteins in D2 adult wild-type, *mars-2(P447V)*, and *nuo-6(qm200)* animals (top). Quantification of the CTC-1/NUO-2 band intensity ratio is shown below in the colored bar graph (*n* = 3; mean and SD shown; 1-way ANOVA with Dunnett's multiple comparisons test). c) Representative fluorescent micrographs showing TMRE staining in D2 adult wild-type and *mars-2[P447V]* animals. d) Scatter plot with bars showing fluorescence intensity quantification of TMRE signal in D2 adult wild-type and *mars-2[P447V]* animals (*n* = 24; mean and SD shown; Welch's *t*-test). e) Stacked bar graph showing proportion of hatched individuals from plated embryos of wild-type and *mars-2[P447V]* strains. f) Stacked bar graph showing the distribution of animals across developmental stages at indicated time points in wild-type and *mars-2[P447V]* strains (*n* = 100). WT, wild-type.

Given that compromising MARS-2 mitochondrial localization activates UPR^mt^, we assessed the mitochondrial localization of the P447V mutant. We found that C-terminally tagging the MARS-2(P447V) protein with GFP rendered *mars-2(P447V)* animals sterile. Therefore, heterozygous animals were used for imaging. Confocal imaging revealed that MARS-2::GFP protein carrying the P447V mutation strongly overlapped with the mitochondrial signal marked by SCPL-4::wrmScarlet (Fig. 6a), with a Manders coefficient of 0.80 (Supplementary Table 1). This result suggests that P447V mutation does not impair the mitochondrial targeting or import of MARS-2.

InterPro domain analysis indicates that the P447V mutation resides within the anticodon-binding domain of the MARS-2 protein. Thus, we hypothesized that P447V mutation reduces its ability to recognize and charge mitochondrial methionine tRNA, thereby compromising mitochondrial protein synthesis. Deficiencies in mitochondrial aaRSs are known to impair oxidative phosphorylation and trigger the UPR^mt^ (Guo et al. 2023). To test this, we performed western blotting to assess levels of a mitochondrial-encoded electron transport chain (ETC) subunit, CTC-1 (MT-CO1 in mammals), and a nuclear-encoded ETC subunit, NUO-2 (NDUFS3 in mammals) in wild-type and *mars-2(P447V)* animals. Because UPR^mt^ can alter the translational landscape of both nuclear and mitochondrial proteins, we included a well-studied UPR^mt^-inducing mutant strain, *nuo-6(qm200)*, as a control (Yang and Hekimi 2010). We observed a significant reduction in the mitochondrially translated ETC subunit CTC-1 in *mars-2(P447V)* animals compared to both wild-type and *nuo-6(qm200)* animals (Fig. 6b and Supplementary Fig. 3). In contrast, the nuclear encoded ETC subunit NUO-2 was not significantly impacted in *mars-2(P447V)* mutant animals. These data suggest that *mars-2(P447V)* compromises mitochondrial protein production.

UPR^mt^ triggered by mitochondrial stress is typically a result of reduced mitochondrial membrane potential (ΔΨ_m_) that impairs ATFS-1 mitochondrial localization (Rolland et al. 2019; Berry et al. 2021). Thus, we next asked whether *mars-2(P447V)* animals have reduced ΔΨ_m_. Using the fluorescent cationic dye Tetramethylrhodamine (TMRE), which accumulates in mitochondria under normal negative ΔΨ_m_, we found that *mars-2(P447V)* animals exhibited a significant decrease in TMRE fluorescence relative to wild-type (Fig. 6c and 6d), suggesting reduced ΔΨ_m_.

To further evaluate population fitness, we assessed hatching and developmental progression. *mars-2(P447V)* animals displayed mild hatching defects, with approximately 12% of the embryos failing to hatch and reach the L2 larval stages (Fig. 6e). In addition, *mars-2(P447V)* animals exhibited a developmental delay of 12 h in reaching adulthood compared to wild-type control (Fig. 6f). Collectively, these findings suggest that the P447V mutation in *mars-2* alters mitochondrial-encoded protein abundance, and loss of ΔΨ_m_. Consequently, these mitochondrial defects likely have negative physiological effects manifesting as mild hatching defects and delayed development, highlighting the essential role of MARS-2 in supporting mitochondrial function and organismal fitness.

### P447V mutation in *mars-2* leads to mitochondrial fragmentation

**Fig. 7.**
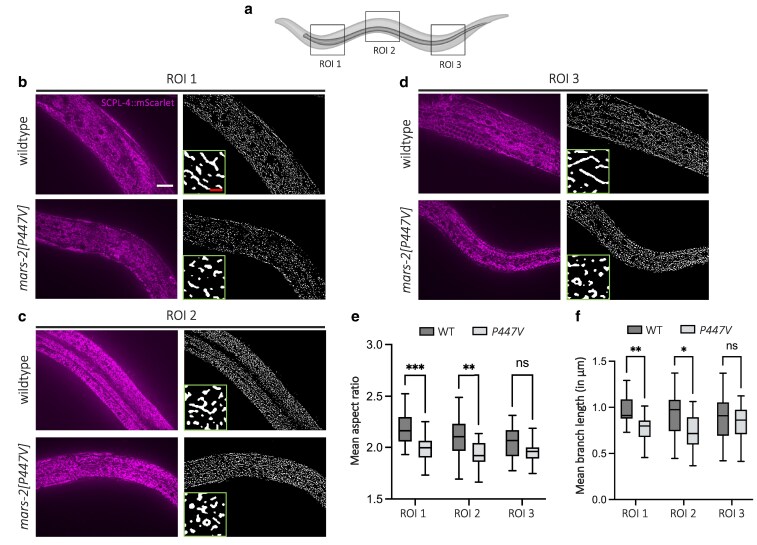
P447V mutation in mars-2 leads to mitochondrial fragmentation. a) Schematic highlighting the 3 regions of interest (ROIs) analyzed in wild-type and *mars-2(P447V)* animals. Representative fluorescent micrographs of D2 wild-type and *mars-2(P447V)* animals showing mitochondrial fluorescence and morphology (left), corresponding mitochondrial masks, and magnified insets (right) for the anterior (b), middle (c), and posterior (d) ROIs. e) Interleaved box and whisker plot showing mean aspect ratio quantification of mitochondria in wild-type and *mars-2(P447V)* animals across the 3 ROIs. f) Interleaved box and whisker plot showing mean branch length quantification of mitochondria in wild-type and *mars-2(P447V)* animals across the 3 ROIs (*n* = 20; median shown as line, whiskers indicate min–max; 2-way ANOVA with Sidak's multiple comparisons test). Scale bars—20 µm (white) and 5 µm (red). WT, wild-type; ROI, region of interest. Created in BioRender. Thapa, B. (2025). https://BioRender.com/4rtvzuy

Given that depletion of mitochondria membrane potential and impaired oxidative phosphorylation are associated with mitochondrial fragmentation (Sprenger and Langer 2019), we next examined mitochondrial morphology in *mars-2(P447V)* animals. To do so, we crossed the *mars-2(P447V)* strain with the WBM1688 (SCPL-4::wrmScarlet) strain and analyzed mitochondrial morphology in 3 distinct regions of the body: anterior, middle, and posterior (Fig. 7a). Animals were imaged using confocal microscopy, and maximum intensity projections were generated for each ROI. Mitochondrial masks were created using Mitochondria Analyzer plugin (Chaudhry et al. 2020) in ImageJ and were used to quantify the mean aspect ratio and mean branch length of mitochondria in each genotype. In the anterior and middle regions (ROI 1 and ROI 2, respectively), *mars-2(P447V)* animals displayed significantly more fragmented mitochondria compared to wild-type animals (Fig. 7b and 7c). The mean aspect ratio was smaller in *mars-2(P447V)* animals, indicating circularity and fragmentation, while the mean branch length was shorter further supporting enhanced mitochondrial fragmentation (Fig. 7e and 7f). Mitochondrial morphology in the posterior region (ROI 3) also trended toward being more fragmented in the mutant animals, though the data did not reach significance (Fig. 7d to 7f). Together, these results demonstrate that *mars-2(P447V)* mutation leads to region-specific mitochondrial fragmentation, particularly in the anterior and middle body regions.

## Discussion

Our study demonstrates that the P447V mutation in the *mars-2* gene represents a loss-of-function allele that activates UPR^mt^. Supporting this model, targeted disruption of the mitochondrial isoform of MARS-2 by substituting the first methionine residue with alanine also led to strong UPR^mt^ activation. Since the P447V mutation occurs in a highly conserved anticodon binding domain of the enzyme (Fig. 1f), it is plausible that this mutation reduces methionine tRNA charging, thereby impairing mitochondrial protein synthesis. Consistent with this hypothesis, we found that *mars-2(P447V)* animals exhibit decreased protein abundance of a mitochondrially encoded ETC subunit.

We did not assess the effects of the P447V mutation on UPR^mt^ specifically on the cytoplasmic isoform (isoform A) of *mars-2* in *C. elegans*. However, substituting 119th methionine residue to alanine specifically disrupts isoform A without affecting the mitochondrial localization of MARS-2 and these animals showed no evidence of UPR^mt^ activation. Given that *mars-1* encodes the established cytoplasmic methionyl tRNA synthetase (Alriyami et al. 2014), the cytoplasmic isoform likely represents a redundant or nonfunctional paralog that does not play a major role in either cytoplasmic or mitochondrial translation. However, we observe a mild suppression of the basal UPR^ER^ reporter in P447V mutant animals. We speculate this observation may reflect mild reduction in cytosolic translation, perhaps a consequence of UPR^mt^. Overall, we predict that the P447V mutation specifically in the cytoplasmic isoform would not be deleterious to mitochondrial function.

Interestingly, UPR^mt^ activation in P447V mutants was confined to the intestine. This tissue-specific response parallels findings in humans, where mitochondrial aaRS mutations predominantly affect high-energy demand tissues such as muscles and the nervous system (Antonellis and Green 2008). The intestinal specificity in *mars-2(P447V)* mutants may therefore reflect intrinsic differences in mitochondrial dynamics or stress susceptibility across tissues. Furthermore, we observed that GFP tagging of MARS-2(P447V) rendered animals sterile, while complete loss of mitochondrial isoform caused developmental arrest, suggesting that the GFP tag partially impairs protein function, with the germline being particularly sensitive to such disruption. Mitochondrial morphology analysis further revealed region-specific fragmentation, with the anterior and middle regions displaying higher fragmentation compared to the posterior. These regional differences likely arise from tissue-specific variation in the sensitivity or intensity of the response to the MARS-2 dysfunction.

In summary, this study establishes a strong link between the P447V mutation in *mars-2* and mitochondrial quality control, implicating impaired global mitochondrial translation as the underlying cause for UPR^mt^ induction, mitochondrial membrane potential loss, and mitochondrial fragmentation. The isoform-specific mutants and point mutants generated here provide valuable tools for dissecting mitochondrial quality control pathways and modeling the pathological consequences of aaRS mutations.

## Supplementary Material

jkaf298_Supplementary_Data

## Data Availability

Strains are available upon request. The authors affirm that all data necessary for confirming the conclusions of the article are present within the article and figures. Supplemental material available at G3 online.
