# Supplementary material for: Identification of an uncharacterized gene as a mitochondrial methionine tRNA synthetase in Caenorhabditis elegans
Source: G3 (Bethesda). 2025 Dec 8;16(2):jkaf298. doi: 10.1093/g3journal/jkaf298 (PMC12869082; doi:10.1093/g3journal/jkaf298)
Supplement: jkaf298_Supplementary_Data [file jkaf298_supplementary_data.zip › Supplemental_Material_Legends_G3-2025-406397.pdf]

Description of supplementary files

Figure S1. MARS-2(P447V)-mediated mitochondrial unfolded protein response is partially dependent on DVE-1 dependent. (a) Representative brightfield and corresponding fluorescent micrographs showing UPR^{mt} reporter activation in day 2 (D2) adult wildtype and *mars-2[P447V]* animals upon control and *dve-1* RNAi for the first generation. Animals were growth arrested for the second generation *dve-1* RNAi and results are not quantified (data not shown). (b) Scatter plot showing fluorescence intensity quantification of the UPR^{mt} reporter in wildtype and *mars-2[P447V]* animals upon control and *dve-1* RNAi normalized to the reporter intensity in wildtype animals on control RNAi (n=24, mean and SD shown, two-way ANOVA with Sidak's multiple comparison test). Scale bar – 200 µm, F.C. – Fold change.

Figure S2. Loss of shorter isoform of MARS-2 (Isoform A) by substituting 119th Methionine to Alanine does not activates mitochondrial unfolded protein response. (a) Representative brightfield and corresponding fluorescent micrographs showing UPR^{mt} reporter activation in D2 adult wildtype and *mars-2[M119A]* animals (second clone). (b) Scatter plot showing fluorescence intensity quantification of the UPR^{mt} reporter in wildtype and *mars-2[M119A]* animals (n = 24, mean and SD shown, unpaired t-test) (c) Scatter plot depicting outline area quantification of wildtype and *mars-2[M119A]* animals (n = 24, mean and SD shown, unpaired t-test). Scale bar – 200 µm, A.U. – Arbitrary units.

Figure S3. Blots for D2 adult wildtype, *mars-2[P447V]*, and *nuo-6[qm200]* animals. Three composite images of chemiluminescent and colorimetric images of the western blots are shown for the three replicates. For each replicate, starting from left, the lanes are labelled. Lane #1 – PageRuler Plus ladder with relevant bands labelled in kDa, Lane #2 – wildtype protein lysate, Lane #3 – *mars-2[P447V]* protein lysate, and Lane #4 – *nuo-6[qm200]* protein lysate.

Table S1. Mander's coefficients showing extent of colocalization between two channels in wildtype, *mars-2[M1A]*, *mars-2[M119A]*, and *mars-2[P447V].*
