# Supplementary material for: Identification of an uncharacterized gene as a mitochondrial methionine tRNA synthetase in Caenorhabditis elegans
Source: G3 (Bethesda). 2025 Dec 8;16(2):jkaf298. doi: 10.1093/g3journal/jkaf298 (PMC12869082; doi:10.1093/g3journal/jkaf298)
Supplement: jkaf298_Supplementary_Data [file jkaf298_supplementary_data.zip › Table_S1_G3-2025-406397.pdf]

Genotype	M1 (channel 1 = SCPL-4::mScarlet)	M2 (channel 2 = MARS-2::GFP)
wildtype	0.57	0.85
mars-2[M1A]	0.32	0.39
mars-2[M1119A]	0.76	0.90
mars-2[P447V]	0.59	0.80
