# Supplementary figures and images for: Identification of an uncharacterized gene as a mitochondrial methionine tRNA synthetase in Caenorhabditis elegans
Source: G3 (Bethesda). 2025 Dec 8;16(2):jkaf298. doi: 10.1093/g3journal/jkaf298 (PMC12869082; doi:10.1093/g3journal/jkaf298)

a

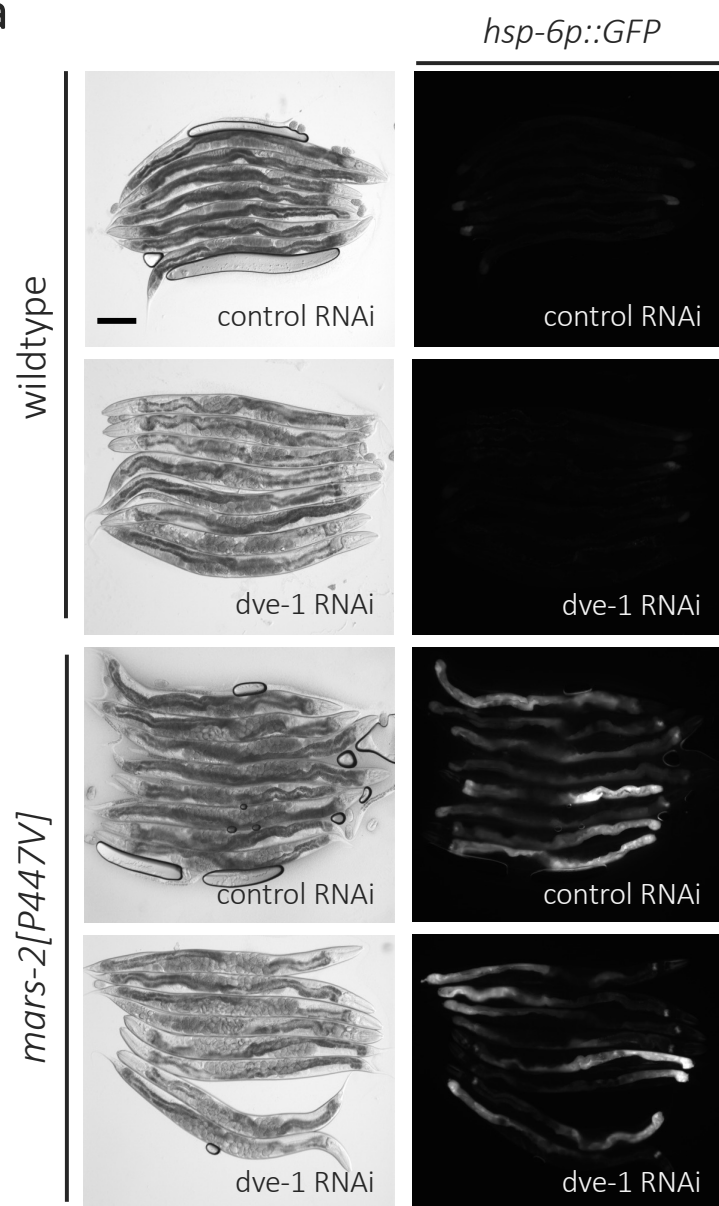

b

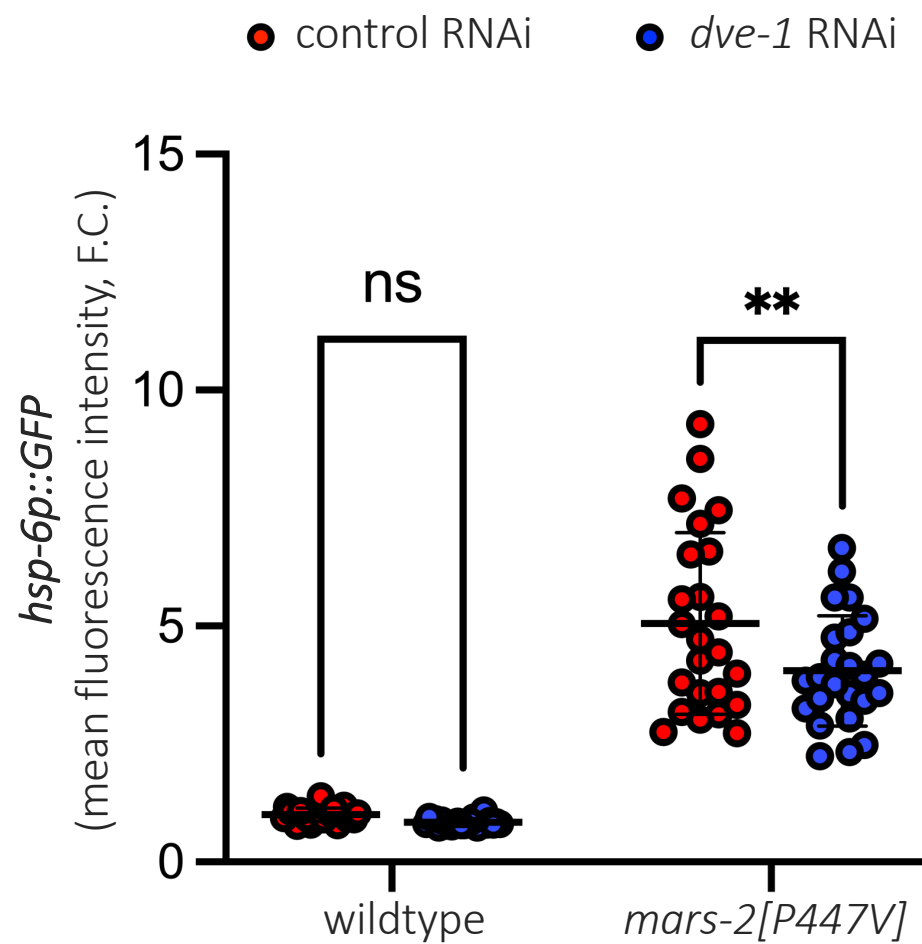

Supplement: jkaf298_Supplementary_Data [file jkaf298_supplementary_data.zip › Figure_S1_G3-2025-406397.pdf]

**a**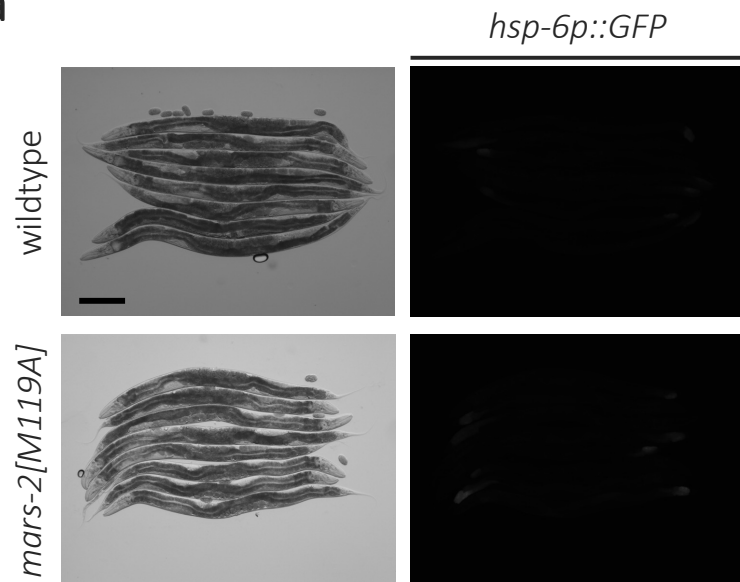**b**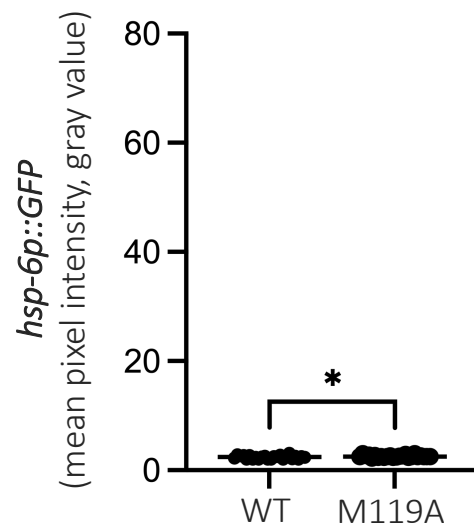**c**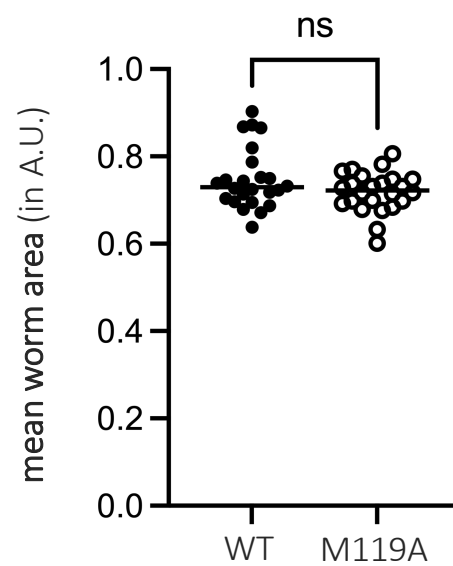

Supplement: jkaf298_Supplementary_Data [file jkaf298_supplementary_data.zip › Figure_S2_G3-2025-406397.pdf]

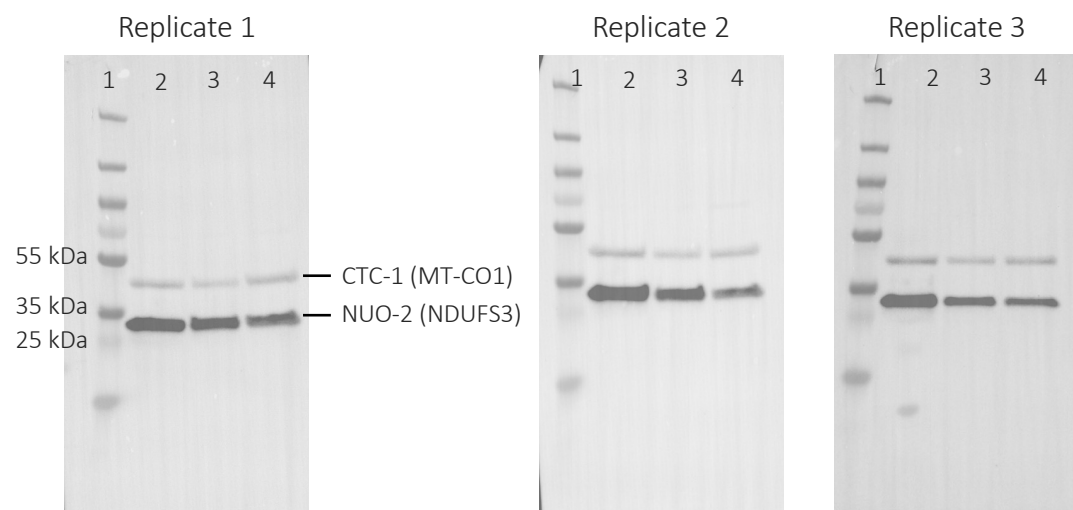

Supplement: jkaf298_Supplementary_Data [file jkaf298_supplementary_data.zip › Figure_S3_G3-2025-406397.pdf]
